# Supplementary material for: Construction of a Diagnostic m7G Regulator-Mediated Scoring Model for Identifying the Characteristics and Immune Landscapes of Osteoarthritis
Source: Biomolecules. 2023 Mar 16;13(3):539. doi: 10.3390/biom13030539 (PMC10046530; doi:10.3390/biom13030539)
Supplement: Supplementary file 1 [file biomolecules-13-00539-s001.zip › Table S2.pdf]

Gene	Primer	Sequence (5'-3')
Hum DCP2	Forward	GATTCCTCAGACAGTGACAATGG
	Reverse	AGTGGTTGCCTGTGCTTTAC
Hum EIF4E2	Forward	ATGGTGGCAAGTGGATTATTCG
	Reverse	CTTGGTCACTGGCAGTCTTATTC
Hum LARP1	Forward	TCCTCCGAGATCACTTCAACA
	Reverse	CTTCATAGTCCTTCACCGTTTCC
Hum SNUPN	Forward	ACGTTCTGGATGTGATGTGCT
	Reverse	CATCACACAGGCTTTCGGGA
Hum GPADH	Forward	GGAAGCTTGTCATCAATGGAAATC
	Reverse	TGATGACCCTTTTGGCTCCC

Table S2: The primer pairs utilized in Real-Time Quantitative PCR.
